# Supplementary material for: A high-resolution mRNA expression time course of embryonic development in zebrafish
Source: eLife. 2017 Nov 16;6:e30860. doi: 10.7554/eLife.30860 (PMC5690287; doi:10.7554/eLife.30860)
Supplement: Supplementary file 6. [file elife-30860-supp6.zip › biolayout-clusters-files/Cluster105-genes.html]

Cluster105


# Cluster105: Genes

| Ensembl ID | Gene Name | Chr | Start | End | Biotype |
| --- | --- | --- | --- | --- | --- |
| ENSDARG00000104034 | arid3b | 25 | 28544755 | 28630862 | protein\_coding |
| ENSDARG00000075211 | chd7 | 2 | 22107585 | 22162635 | protein\_coding |
| ENSDARG00000002082 | csnk2a1 | 16 | 31478830 | 31494580 | protein\_coding |
| ENSDARG00000000394 | matr3l1.1 | 14 | 24782537 | 24802743 | protein\_coding |
| ENSDARG00000061052 | nipblb | 10 | 36532102 | 36603370 | protein\_coding |
| ENSDARG00000025285 | panx1a | 15 | 2512322 | 2526362 | protein\_coding |
| ENSDARG00000016664 | rnf20 | 14 | 20769850 | 20801098 | protein\_coding |
| ENSDARG00000058089 | rprd2a | 19 | 8975027 | 9007650 | protein\_coding |
| ENSDARG00000101808 | si:ch211-271b14.1 | 5 | 67157884 | 67182608 | protein\_coding |
| ENSDARG00000037965 | srrm2 | 3 | 32499658 | 32518564 | protein\_coding |
| ENSDARG00000040184 | syncrip | 20 | 2714688 | 2728646 | protein\_coding |
| ENSDARG00000042484 | tle2 | 22 | 21593143 | 21642854 | protein\_coding |
